# Activation of Protease-Activated Receptor 2-Mediated Signaling by Mast Cell Tryptase Modulates Cytokine Production in Primary Cultured Astrocytes

**DOI:** 10.1155/2013/140812

**Published:** 2013-06-02

**Authors:** Xiaoning Zeng, Shu Zhang, Luwei Xu, Haiwei Yang, Shaoheng He

**Affiliations:** ^1^Clinical Research Centre, the First Affiliated Hospital of Nanjing Medical University, 300 Guangzhou Road, Nanjing, Jiangsu 210029, China; ^2^Department of Urology, Nanjing First Hospital Affiliated to Nanjing Medical University, 68 Changle Road, Nanjing, Jiangsu 210006, China

## Abstract

Protease-activated receptor 2 (PAR-2), which is abundantly expressed in astrocytes, is known to play major roles in brain inflammation. However, the influence of the natural agonist of PAR-2, tryptase, on proinflammatory mediator releasedfrom astrocytes remains uninvestigated. In the present study, we found that tryptase at lower concentrations modestly reduced intracellular ROS production but significantly increased IL-6 and TNF-**α** secretion at higher concentrations without affecting astrocytic viability and proliferation. The actions of tryptase were alleviated by specific PAR-2 antagonist FSLLRY-NH2 (FS), indicating that the actions of tryptase were via PAR-2. PI3K/AKT inhibitor LY294002 reversed the effect of tryptase on IL-6 production, whereas inhibitors specific for p38, JNK, and ERK1/2 abolished the effect of tryptase on TNF-**α** production, suggesting that different signaling pathways are involved. Moreover, tryptase-induced activation of MAPKs and AKT was eliminated by FS, implicating that PAR-2 is responsible for transmitting tryptase biosignals to MAPKs and AKT. Tryptase provoked also expression of TGF-**β** and CNTF in astrocytes. The present findings suggest for the first time that tryptase can regulate the release of cytokines from astrocytes via PAR-2-MAPKs or PAR-2-PI3K/AKT signaling pathways, which reveals PAR-2 as a new target actively participating in the regulation of astrocytic functions.

## 1. Introduction

As a unique family of G protein-coupled receptors, newfound protease-activated receptors (PARs) are widely expressed on the cells in central nervous system (CNS), including neurons and glial cells [1], regulating cell responses to extracellular serine proteases as cell surface sensors and contributing extensively to the regulation of homeostasis as well as to the dysfunctional responses of these cells required for progression of cerebral diseases [2]. Among the four PARs identified to date, PAR-2 is a unique one activated by trypsin and mast cell tryptase while others (PAR-1, -3, and -4) activated by thrombin [3]. The role of PAR-2, which is distributed extensively throughout the nervous system (including CNS and peripheral nervous system), has been principally investigated in peripheral nervous system, where it is known to play major roles in injury, inflammation, neuronal signaling, and nociception [4, 5]. And the physiological role of PAR-2 in CNS remains unclear but its activation has been shown to increase intracellular Ca^2+^ levels in both neurons and astrocytes [6, 7] as well as trigger the release of gliotransmitters such as GRO/CINC-1 [8–10] and nitric oxide [11]. Recent group of evidence have revealed that PAR-2 contributes to neuroprotection and/or neurodegeneration in the brain under pathological conditions [12–15]. Therefore, PAR-2 has been suggested to be a novel therapeutic target for the treatment of brain disorders.

Tryptase, the major secretory protein of mast cells, is the natural agonist of PAR-2 and can stimulate peripheral mononuclear cells to secrete tumor necrosis factor-alpha (TNF-*α*) and interleukin-6 (IL-6) [16] to induce widespread inflammation [17]. Although mast cells typically reside at barrier sites of the body such as the intestinal mucosa and blood brain barrier (BBB) [18], Silverman et al. found that mast cells can rapidly penetrate brain blood vessels and migrate into the neural parenchyma [19], implying an interaction between mast cells and nerve tissue cells. As the most abundant cells in brain parenchyma, astrocytes play pivotal roles in BBB integrity and CNS function such as synapse formation [20], communication [21], cerebrovascular tone [22], adult neurogenesis [23], as well as neuroimmune [24]. Since PAR-2 is widely expressed in astrocytes and is recognized for the modulatory properties of neuroinflammation and neurodegeneration such as multiple sclerosis [25], its contribution to astrocytic functions remains to be elucidated. In the present study, we investigated the consequence of tryptase stimulation on (1) the astrocytic survival and proliferation; (2) the production of IL-6, TNF-*α*, and reactive oxygen species (ROS); (3) the involvement of MAPKs and PI3K/AKT pathways in PAR-2 activation; (4) the levels of potent neural cytokines transforming growth factor-*β* (TGF-*β*) and ciliary neurotrophic factor (CNTF) generated by astrocytes.

## 2. Materials and Methods

### 2.1. Reagents

Dulbecco's modified Eagle's medium (DMEM) and fetal bovine serum (FBS) were purchased from Gibco-BRL (Grand Island, NY, USA). Poly-D-lysine, tryptase, SB203580, PD98059, SP600125, and LY294002 were purchased from Sigma-Aldrich (St. Louis, MO, USA). PAR-2 inhibitor FS were synthesized by CL Bio-Scientific Inc. (Xi An, China). Dojindo Cell Counting Kit-8 was purchased from Sigma-Aldrich (St. Louis, MO, USA). Rat IL-6 Immunoassay Kit and Rat TNF-*α* Immunoassay Kit were obtained from R&D Systems, Inc. (Minneapolis, MN, USA). LIVE green reactive oxygen species detection kit was purchased from Molecular Probes Invitrogen (Carlsbad, CA, USA). Specific glial fibrillary acid protein (GFAP) antibody (a marker for astrocytes) was purchased from Sigma-Aldrich (St. Louis, MO, USA). Specific monoclonal antibodies against p38, phospho-p38, SAPK/JNK (c-JUN N-terminal kinase), phospho-SAPK/JNK, p44/42 MAPK (extracellular regulated protein kinases, ERK), phospho-p44/42 MAPK (phospho-ERK) and AKT, and phospho-AKT were obtained from Cell Signaling (Beverly, MA, USA). Specific polyclonal antibodies against TGF-*β* and CNTF were purchased from Abcam (Cambridge, MA, UK).

### 2.2. Primary Astrocyte Cultures

Confluent primary astrocyte cultures were prepared from Sprague-Dawley rats as previously described with slight modification. All animal procedures were performed according to the NIH Guide for Animal Care and approved by the institutional animal care and use committee. Briefly, postnatal (P1-P2) rats were killed by rapid decapitation, cerebral cortices were triturated and cells were plated on poly-D-lysine precoated culture flasks in DMEM, containing 10% FBS, 100 U/mL penicillin, and 100 mg/mL streptomycin. Cultures were maintained at 37°C in a humidified atmosphere of 5% CO_2_/95% air. Culture medium was replaced 24 h later and then changed every 2-3 days. After reaching a confluent monolayer of cells (10–14 days), microglia were eliminated from astrocytes by shaking off for 5 h at 100 r.p.m. and astrocytes were replated in poly-D-lysine coated culture dishes, 96-well or 6-well plates. The enriched astrocytes were >98% pure as determined by astrocytic marker GFAP. 

### 2.3. Cell Proliferation Assay

Cell viability was measured by conversion of Dojindo's highly water-soluble tetrazolium salt WST-8 to a yellow-colored water-soluble formazan (CCK8 assay). The amount of formazan dye generated by the activity of mitochondrial dehydrogenases in cells is directly proportional to the number of living cells. CCK8 is more sensitive than the 3-(4,5-dimethylthiazol-2-yl)-2,5-diphenyltetrazolium bromide assay [26]. Cells were collected and seeded in 96-well plates at a density of 10^5^ cells/cm^2^. After incubation for 48 h, cells were exposed to fresh medium containing various concentrations of tryptase (0.001, 0.01, 0.1, 1, and 10 *μ*g/mL) at 37°C for further 24 h. Then, 20 *μ*L of CCK8 solution in PBS was added to each well and the plates were incubated for an additional 2 h. The optical density of each well was measured using a microculture plate reader at a 450 nm wavelength.

### 2.4. Intracellular Reactive Oxygen Species Assay

The production of intracellular ROS was analyzed by 2′,7′-dichlorodihydrofluorescein (DCFH) oxidation. The 2′,7′-dichlorodihydrofluorescein diacetate (DCFH-DA) reagent passively enters cell where it is deacetylated by esterase to nonfluorescent DCFH. Inside the cell, DCFH reacts with ROS to form 2′,7′-dichlorofluorescein (DCF), the fluorescent product. For this assay, 10 mM of DCFH-DA was dissolved in DMSO and was diluted 500-fold in HBSS to give 20 *μ*M of DCFH-DA. Enriched-astrocyte cultures seeded (5 × 10^4^) in 96-well plates were then exposed to DCFH-DA for 1 h at 37°C in dark, followed by treatment with HBSS containing various concentrations of tryptase for 2 h. After being rinsed twice with PBS, green fluorescence from DCF in cells was measured in the FL1 Log channel through a 525-nm band-pass filter on the Coulter EPICS XL/X1-MCL (Beckman Coulter Company, Miami, FL, USA).

### 2.5. IL-6 and TNF-*α* Assay

The amount of IL-6 and TNF-*α* in the culture medium was measured with commercial ELISA kits from R&D Systems, respectively.

### 2.6. Western Blot Analysis

Cells were collected and homogenized in 200 *μ*L lysing buffer. After incubation for 20 min on ice, cell lysate was centrifuged at 12,000 g at 4°C for 10 min and protein concentration in the extracts was determined by the Bradford assay. Proteins in cell extracts were denatured with sodium dodecyl sulfate (SDS) sample buffer and separated by 10% SDS-polyacrylamide gel at 80 V for 2 h. Then proteins were electrotransferred to nitrocellulose membranes at 300 mA for various time points by using a Bio-Rad miniprotein-III wet transfer unit. The membranes were blocked with 5% BSA dissolved in Tris-buffered saline with Tween 20 (TBST) (pH 7.5, 10 mM Tris-HCl, 150 mM NaCl and 0.1% Tween 20) at room temperature for 1 h. This was followed by incubating the membranes with different antibodies (anti-p38, -phospho-p38; -JNK, -phospho-JNK; -ERK, -phospho-ERK; -AKT, -phospho-AKT; -TGF-*β* at 1 : 800 dilution and -CNTF at 1 : 1500 dilution) overnight at 4°C, and finally incubated with a horseradish peroxidase-conjugated anti-rabbit IgG for 1 h at room temperature. Protein bands on the membranes were visualized by an enhanced chemiluminescence kit.

### 2.7. Statistical Analysis

All values shown are presented as means ± SEM. The significance of the difference between control and samples treated with various drugs was determined by one-way ANOVA followed by the post-hoc least significant difference test. Differences were considered statistically significant at *P* < 0.05. 

## 3. Results

### 3.1. Tryptase Had No Effect on the Astrocytic Viability

**Figure 1:**
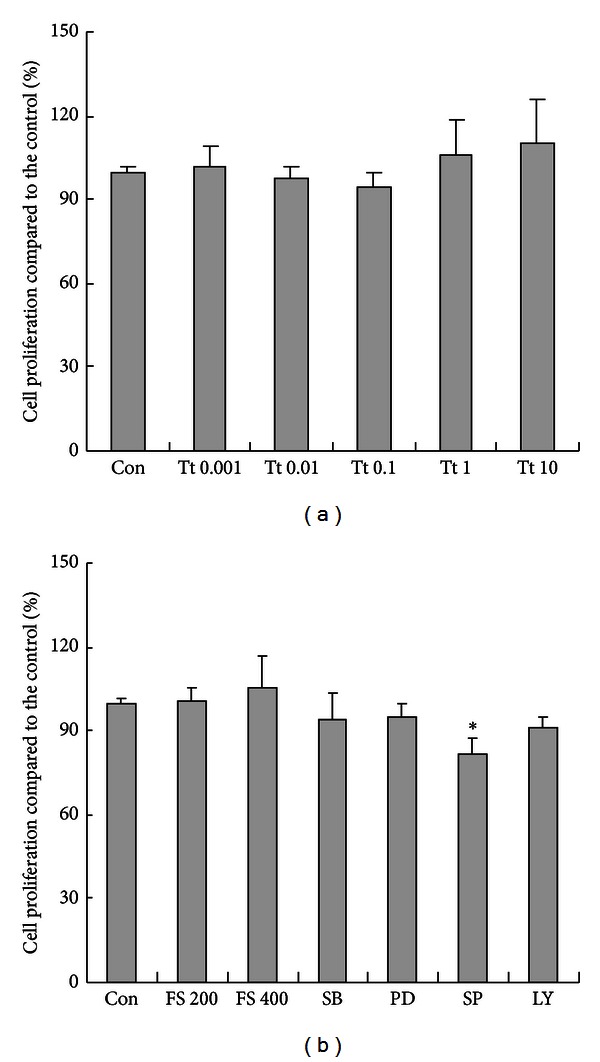
Effects of tryptase (Tt) and specific inhibitor of PAR-2 or MAPKs on the viability of astrocytes. For the dose-dependent studies astrocytes were treated with either culture medium only or various concentrations of tryptase (0.001, 0.01, 0.1, 1, and 10 *μ*g/mL), FSLLRY-NH2 (FS, 200 or 400 *μ*M), SB203580 (SB, 20 *μ*M), PD98059 (PD, 20 *μ*M), SP600125 (SP, 20 *μ*M) and LY294002 (LY, 20 *μ*M) for 24 h. Data are presented as the mean ± SEM in triplicate on four separate occasions. *P < 0.05 versus control groups (Con).

Cell survival measured by CCK8 analysis revealed that incubation with different dose of tryptase (0.001, 0.01, 0.1, 1, and 10 *μ*g/mL) for 24 h had no significant effect on astrocytic viability and proliferation. No impact was either observed in specific PAR-2 inhibitor FS (200 or 400 *μ*M), p38 inhibitor SB203580 (20 *μ*M), JNK inhibitor SP600125 (20 *μ*M), ERK1/2 inhibitor PD98059 (20 *μ*M) and PI3K/AKT inhibitor LY294002 (20 *μ*M) (Figure 1).

### 3.2. Tryptase Inhibits ROS Production in Primary Cultured Astrocytes via PAR-2

**Figure 2:**
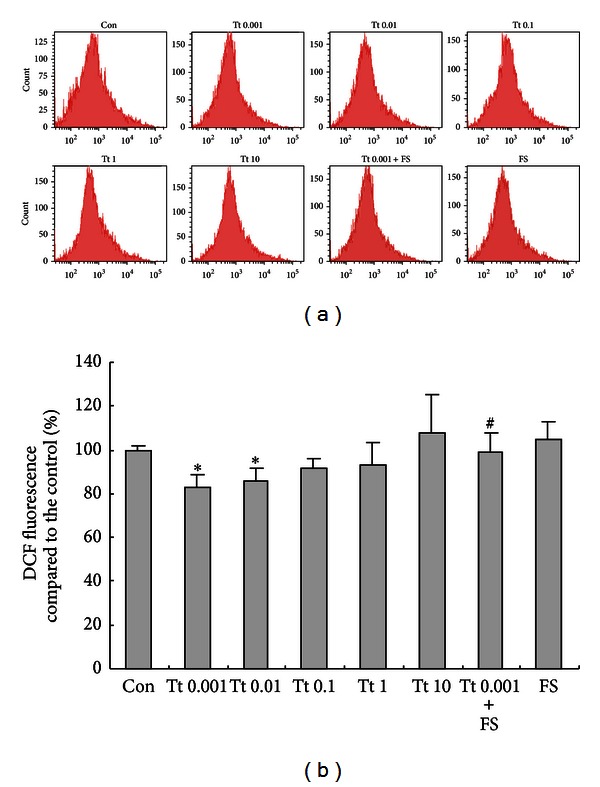
Effects of tryptase (Tt) on the generation of reactive oxygen species (ROS) from astrocytes. Astrocytes were exposed to different concentrations of tryptase (0.001, 0.01, 0.1, 1, and 10 *μ*g/mL) in the presence or absence of FSLLRY-NH2 (FS, 400 *μ*M), an antagonist of tryptase at 37°C for 2 h. ROS was presented as changes of DCF fluorescence. Data are presented as the mean ± SEM analyzed from three independent sets of samples. *P < 0.05 versus control group (Con), ^#^ *P* < 0.05 versus corresponding tryptase treatment group.

The results of the DCF assay indicated that incubation with tryptase at low concentrations (0.001 and 0.01 *μ*g/mL) for 2 h modestly inhibited the intracellular levels of ROS, which was abolished by FS (400 *μ*M) (Figure 2). The fluorescence of DCF in cells decreased to 83% (0.001 *μ*g/mL group) and 86% (0.01 *μ*g/mL group) of that in the control, and specific PAR-2 inhibitor FS diminished the effects of tryptase (0.001 *μ*g/mL) on ROS generation in astrocytes, implying that activation of PAR-2 at low concentrations of tryptase is responsible for the inhibition of ROS production.

### 3.3. Tryptase Regulated IL-6 and TNF-*α* Secretion from Primary Cultured Astrocytes via PAR-2

**Figure 3:**
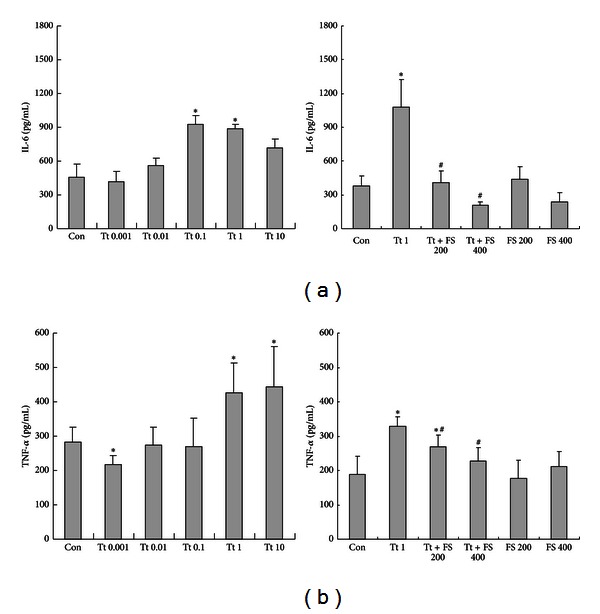
Effects of tryptase (Tt) on the secretion of IL-6 (a) and TNF-*α* (b) from astrocytes. Astrocytes were exposed to different concentrations of tryptase (0.001, 0.01, 0.1, 1, and 10 *μ*g/mL) in the presence or absence of FSLLRY-NH2 (FS, 200 or 400 *μ*M), an antagonist of tryptase at 37°C for 24 h before culture supernatant being collected. Data are presented as the mean ± SEM of four independent experiments. *P < 0.05 versus control groups (Con), ^#^ *P* < 0.05 versus corresponding tryptase treatment groups.

As shown in Figure 3, incubation with tryptase at the dose of 0.001, 0.01, 0.1, 1, and 10 *μ*g/mL for 24 h produced a concentration dependent increase in IL-6 secretion from primary cultured astrocytes with a minimum effective dose of 0.1 *μ*g/mL (Figure 3(a)). Meanwhile, tryptase (1 and 10 *μ*g/mL) also increased TNF-*α* secretion at a minimum effective dose of 1 *μ*g/mL (Figure 3(b)), but modestly inhibited TNF-*α* secretion at the dose of 0.001 *μ*g/mL. PAR-2 inhibitor FS (200 and 400 *μ*M) was able to diminish tryptase (1 *μ*g/mL) induced IL-6 and TNF-*α* increase but itself alone (200 and 400 *μ*M) failed to affect IL-6 and TNF-*α* secretion from astrocytes (Figures 3(a) and 3(b)), suggesting that tryptase is able to modulate the secretion of IL-6 and TNF-*α* from astrocytes via PAR-2.

### 3.4. Tryptase Regulated TNF-*α* but Not IL-6 Secretion via MAPKs from Primary Cultured Astrocytes

**Figure 4:**
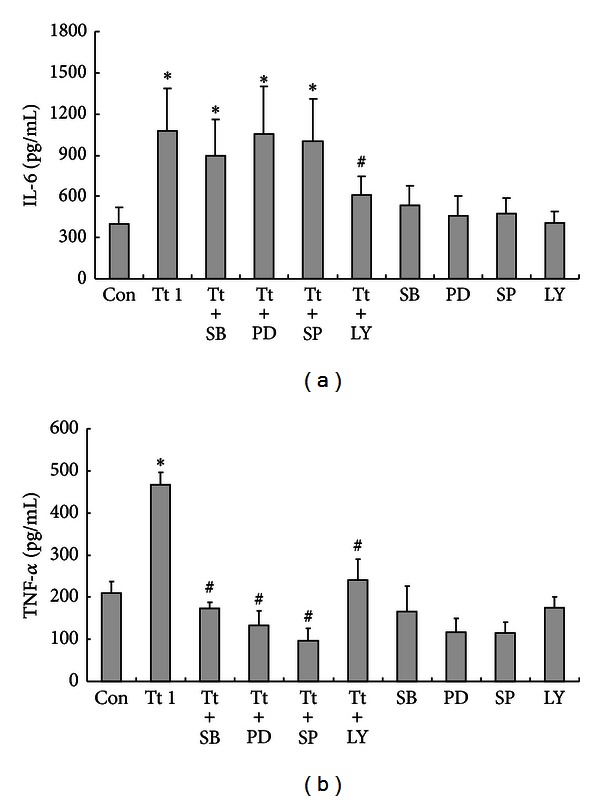
Effects of inhibitors of cell signaling pathways on tryptase (Tt)-induced secretion of IL-6 (a) and TNF-*α* (b) from astrocytes. Astrocytes were exposed to 1 *μ*g/mL of tryptase in the presence or absence of SB203580 (SB, 20 *μ*M), PD98059 (PD, 20 *μ*M), SP600125 (SP, 20 *μ*M), and LY294002 (LY, 20 *μ*M) at 37°C for 24 h before culture supernatant being collected. Data are presented as the mean ± SEM of four independent experiments. *P < 0.05 versus control groups (Con), ^#^ *P* < 0.05 versus corresponding tryptase treatment groups.

To investigate the involvement of MAPKs and PI3K/AKT signaling pathways in the IL-6 and TNF-*α* secretion, we used pharmacological inhibitors of MAPKs and PI3K/AKT. SB203580 is a pyridinyl imidazole compound which acts as a competitive inhibitor of ATP binding on the p38 kinase and thus serves as a specific inhibitor of p38 MAPKs. PD98059 is a potent, selective, and cell-permeable inhibitor of MEK1, which results in inhibition of the phosphorylation and activation of ERK1/2. SP600126 is a potent, selective, reversible, and cell-permeable inhibitor of JNK, a Ser/Thr kinase that phosphorylates c-jun. LY294002 is a potent, selective, cell permeable, and specific inhibitor of PI3K/AKT. As shown in Figure 4, SB203580 (20 *μ*M), PD98059 (20 *μ*M), SP600125 (20 *μ*M), and LY294002 (20 *μ*M) alone did not have impacts on the secretion of IL-6 and TNF-*α* from astrocytes. tryptase (1 *μ*g/mL) induced IL-6 increase was reversed by LY294002, but not by inhibitors of MAPKs (SB203580, PD98059, SP600125) (Figure 4(a)). However, tryptase (1 *μ*g/mL) induced TNF-*α* increase was reversed by SB203580, PD98059, SP600125, and partially by LY294002 (Figure 4(b)), indicating that PI3K/AKT signaling pathway contributes to the secretion of TNF-*α* and IL-6 induced by tryptase, respectively, whereas MAPKs signaling pathway is involved in the tryptase induced secretion of TNF-*α*, but not IL-6.

### 3.5. Tryptase Activated MAPKs and PI3K/AKT in Primary Cultured Astrocytes via PAR-2

**Figure 5:**
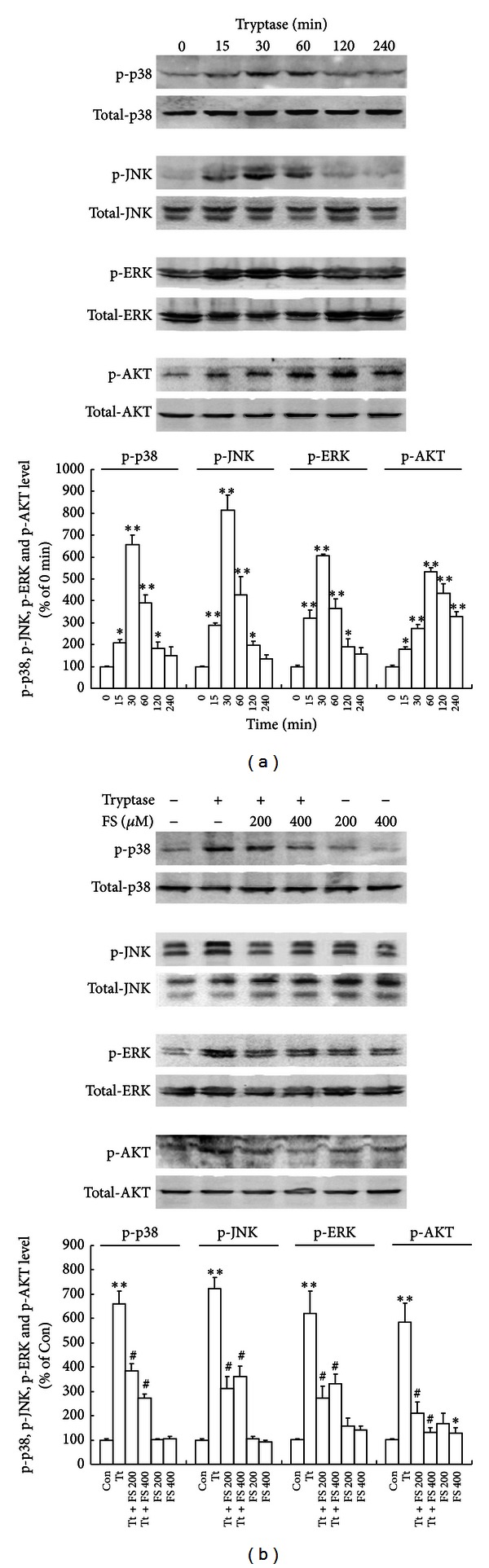
Effects of tryptase (Tt) on the activation of MAPKs and PI3K/AKT. (a) Time courses of tryptase activated p38, JNK, ERK, and AKT, which was assessed by increased phosphorylation of tyrosine residues of these kinases. Astrocytes were incubated with 1 *μ*g/mL of tryptase for the indicated periods (0, 15, 30, 60, 120, and 240 min). (b) FSLLRY-NH2 (FS, 200 or 400 *μ*M) alleviated tryptase (1 *μ*g/mL) induced MAPKs and PI3K/AKT activation. Astrocytes were incubated with 1 *μ*g/mL of tryptase in the presence or absence of FS, an antagonist of tryptase at 37°C for 30 min. Activated p38 (p-p38), JNK/SAPK (p-JNK), ERK (p-ERK), and AKT (p-AKT) species were detected by immunoblot analysis with antibodies specific for the phosphorylated forms of each kinase. The amount of protein loaded in each lane was confirmed by measuring the amount of p38, JNK, ERK, and AKT reacted to the antibody against the unphosphorylated form of each kinase. This is a representative experiment independently performed three times. **P < 0.01, *P < 0.05 versus control groups (Con), ^#^ *P* < 0.05 versus corresponding tryptase treatment groups.

In order to further understand the actions of tryptase on astrocytes, we examined the effects of tryptase on phosphorylation of cell signaling molecules. Tryptase at 1 *μ*g/mL activated p38 MAPK, JNK, ERK (p44/42), and AKT, which was confirmed by increased phosphorylation of tyrosine residues of these kinases as determined by Western blot analysis. The time course experiments showed that treatment with tryptase (1 *μ*g/mL) led to a rapid and transient phosphorylation of MAPKs and AKT with the peak levels of phospho-p38, phospho-JNK, and phosphor-ERK (p44/42) occurring at 30 min, and phospho-AKT at 60 min, respectively (Figure 5(a)). Astrocytes were pretreated with PAR-2 antagonist FS (200 and 400 *μ*M) for 30 min and then exposed to tryptase (1 *μ*g/mL) for another 30 min or 60 min for detecting the phosphorylation of MAPKs and AKT, respectively. PAR-2 antagonist FS alleviated tryptase-induced MAPKs activation but itself alone had no effect on the phosphorylation of MAPKs and AKT in astrocytes (Figure 5(b)), indicating that activation of PAR-2 by tryptase might be responsible for the activation of MAPKs and PI3K/AKT. 

### 3.6. Tryptase Enhanced TGF-*β* and CNTF Expression in Primary Cultured Astrocytes

**Figure 6:**
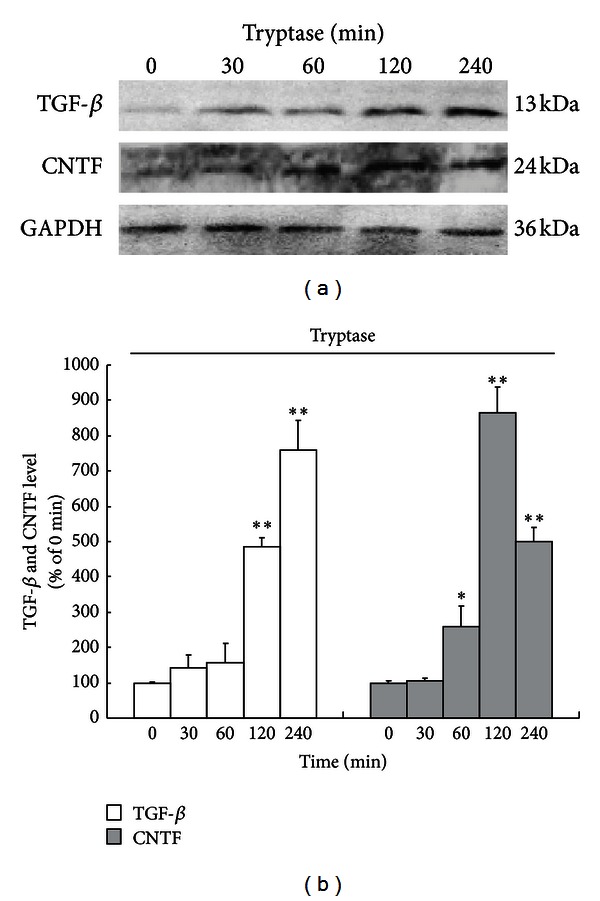
Time courses of tryptase (Tt) on the expression of transforming growth factor-*β* (TGF-*β*) and ciliary neurotrophic factor (CNTF) in astrocytes. (a) Tryptase at 1 *μ*g/mL was incubated with astrocytes at 37°C for 0, 30, 60, 120, and 240 min, respectively. Protein levels were detected by immunoblot analysis with specific antibodies. Western blots at the top of each panel are from a typical experiment. (b) Bar graphs are the quantified results expressed as mean ± SEM of TGF-*β* and CNTF levels from three independent experiments. **P < 0.01, *P < 0.05 versus 0 min.

The time course study (incubation with 1 *μ*g/mL tryptase for 0, 30, 60, 120, 240 min) showed that tryptase significantly promoted TGF-*β* expression in astrocytes, which began at 2 h and lasted at least until 4 h. However, an increase of CNTF expression induced by tryptase (1 *μ*g/mL) initiated rapidly at 1 h following incubation and lasted at least until 4 h (Figure 6). The enhancement in TGF-*β* and CNTF levels indicates that tryptase can induce endogenous production of TGF-*β* and CNTF, which probably contributes to neuroprotection. 

## 4. Discussion

Astrocytes, microglia, and endothelial cells are involved in the intracerebral immune response where they act, in part, by secreting cytokines, chemokines, neurotrophic or neurotoxic factors [27]. Among them, astrocytes play an essential role in neuronal life-support and contribute to the BBB. They also act as neuroprotectors by secreting neurotrophins and release potentially toxic inflammatory molecules [28]. So in the present work, we put our focus on astrocytes with an emphasis on its potential role in the cytokine production involved in neuroimmune processes.

In this study, we first found that tryptase is able to regulate cytokines release from astrocytes without affecting astrocytic viability and proliferation via PAR-2-MAPKs or PAR-2-PI3K/AKT signaling pathway, revealing a novel profile of PAR-2 as a new target in the regulation of astrocytic function.

Tryptase, a major secretory protein of human mast cells, is an endogenous peptide which specifically activates PAR-2. Incubation with tryptase at low concentrations (0.001 and 0.01 *μ*g/mL) modestly inhibited the intracellular levels of ROS in astrocytes, indicating this protease is a potent factor to modulate astrocytes-derived ROS. The specific antagonist peptide of PAR-2 FS diminished the effect of tryptase on ROS generation, implying that the inhibition of the intracellular ROS by tryptase might at least partially be responsible for the activation of PAR-2. In the brain, ROS exerts a key role in normal physiological functions and neuroimmune responses [26, 29], and astrocytes are the important sources of ROS. Imbalance in the level of ROS has been shown to be a causative factor in numerous pathologies such as ischemia/reperfusion injury and degenerative diseases [29, 30]. Considerable amount of evidence suggests oxidative stress induced by astrocytes-derived ROS is a crucial contributor to neurodegeneration [31, 32]. Our current observation implicates that moderate stimulation of PAR-2 existed in astrocytes might regulate astrocytes-derived ROS and supplies a new option for the management of CNS functions.

IL-6 is a pleiotropic cytokine involved in the regulation of inflammatory and immunological responses, acute phase protein production, and hematopoiesis [33]. There is increasing evidence supporting a role for the IL-6 receptor family in CNS development, as well as during neurodegeneration and regeneration [34]. Like many cytokines, IL-6 may have distinct physiological effects at different concentrations and in different biological contexts [35]. Like IL-6, TNF-*α* now appears also to play an important role in neural plasticity and neurorepair [36–38] in addition to its well established function as a proinflammatory cytokine. Here we presented data investigating the consequence of PAR-2 activation by various concentrations of tryptase on IL-6 and TNF-*α* secretion from astrocytes. We found that tryptase administration produced a concentration dependent increase in IL-6 and TNF-*α* secretion with a minimum effective dose of 0.1 *μ*g/mL and 1 *μ*g/mL, respectively, suggesting that the stimulation of IL-6 generation by tryptase is more sensitive than that of TNF-*α* in astrocytes, which is distinct from the data obtained from microglia [39]. Since neuroinflammation could generate an environment detrimental for repair, alternatively it could also create an environment permissive for neurorepair [40], our findings implicate that mast cell tryptase is able to regulate the activity of astrocytes and the levels of neuroinflammatory proteins released from astrocytes such as IL-6 and TNF-*α* so as to modulate the balance between neuroinflammation and neurorepair [33].

Moreover, tryptase-induced IL-6 and TNF-*α* release from astrocytes appeared to rely on the activation of PAR-2 as a specific antagonist peptide of PAR-2 FS was able to block the action of tryptase on astrocytes. PAR-2 was identified to be expressed abundantly on astrocytes [6], PAR-2 activation in astrocytes has been demonstrated to play a key modulatory role in diverse pathological conditions [15, 41, 42], but the underlying mechanisms remain to be clarified. The intracellular downstream MAPKs have been demonstrated to be involved in the regulation of astrocytic function including the production of neuroinflammatory factors in astrocytes. Indeed, our results showed that the intracellular signaling mechanisms that mediate tryptase-induced TNF-*α* release rather than IL-6 release from astrocytes are p38, JNK, and ERK dependent, as SB203580, PD98059, and SP600125 all significantly eliminated TNF-*α* release. As well, tryptase-induced TNF-*α* increase was partly due to PI3K/AKT signaling. Similarly, PI3K/AKT signaling is also responsible for the enhanced production of IL-6, which is consistent with the finding previously reported [43]. Thus, our data provide new evidence that PI3K/AKT signaling pathway contributes to the secretion of TNF-*α* and IL-6 induced by tryptase, whereas MAPKs signaling pathway is involved in the tryptase induced secretion of TNF-*α*, but not IL-6.

In addition, since TGF-*β* and CNTF are potent neural cytokines with very low expression predominantly by astrocytes [44] and both cytokines play very important roles in the modulation of CNS function including neuroinflammation and neurorepair, the levels of TGF-*β* and CNTF expressed in astrocytes were determined in our study. We found that tryptase administration significantly upregulated TGF-*β* and CNTF expression in astrocytes initiated at 2 h and 1 h, respectively, implying that tryptase is as well potent to increase endogenous levels of TGF-*β* and CNTF which probably contribute to neuroprotection. CNTF is almost exclusively produced in the nervous system and can rescue various types of adult CNS neurons in disease models [45–47]. TGF-*β* performs a critical function in nervous system development and repair [48–50]. Both of them are closely implicated in neurodevelopment and neurorepair. Our results not only reveal tryptase as a potent factor to regulate TGF-*β* and CNTF production in astrocytes but may also provide a novel therapeutic option to neurological disorders. Obviously, more detailed work is required to address the issue further.

In conclusion, to our knowledge, this is the first study to demonstrate the ability of mast cell tryptase in modulation of astrocytic activation and astrocytes-derived cytokine production via PAR-2. The implicated signaling mechanisms that mediate the actions of tryptase might be PAR-2-MAPKs or PAR-2-PI3K/AKT pathways, providing a novel profile of PAR-2 as a new target in the regulation of astrocytic function.
